# How Physical Activity Affects Knee Cartilage and a Standard Intervention Procedure for an Exercise Program: A Systematic Review

**DOI:** 10.3390/healthcare10101821

**Published:** 2022-09-21

**Authors:** Luca Petrigna, Federico Roggio, Bruno Trovato, Marta Zanghì, Claudia Guglielmino, Giuseppe Musumeci

**Affiliations:** 1 Department of Biomedical and Biotechnological Sciences, Section of Anatomy, Histology and Movement Science, School of Medicine, University of Catania, Via S. Sofia n°97, 95123 Catania, Italy; 2 Sport and Exercise Sciences Research Unit, Department of Psychology, Educational Science and Human Movement, University of Palermo, Via Giovanni Pascoli 6, 90144 Palermo, Italy; 3 Research Center on Motor Activities (CRAM), University of Catania, Via S. Sofia n°97, 95123 Catania, Italy; 4 Department of Biology, College of Science and Technology, Temple University, Philadelphia, PA 19122, USA

**Keywords:** exercise, physical fitness, osteoarthritis, prevention, health

## Abstract

(1) Background: Cartilage degeneration with the natural aging process and the role of physical activity on cartilage wellness is still not clear. The objective of the present review was to understand how different physical activity interventions affect the cartilage and to propose a Standard Operating Procedure for an exercise program to maintain knee joint health; (2) Methods: Articles were collected on three different electronic databases and screened against the eligibility criteria. Results were collected in tables and the main outcomes were discussed narratively; (3) Results: A total of 24 studies have been included after the screening process and aerobic, strength, flexibility, postural balance, and mobility interventions were detected. Different protocols and types of interventions were adopted by the authors; (4) Conclusions: Physical activity interventions have mainly positive outcomes on cartilage structure, but the protocols adopted are different and various. A Standard Operating Procedure has been proposed for a physical intervention focalized on cartilage wellness that could be adopted as an intervention in the clinical setting. Furthermore, the creation of a standardized protocol wants to help scientific research to move in the same direction.

## 1. Introduction

The natural process of aging has an impact on articular cartilage with chondrocyte loss and a decline in metabolic response, alterations to the matrix and synovial tissue composition, and impairing the ability to maintain and repair these tissues [1]. Chondrocyte senescence contributes to cartilage degeneration, characterized by oxidative stress and the production of cytokines causing the so-called stress-induced senescent state [2]. A sedentary lifestyle with the consequent absence of loading for the cartilage accelerates the progression of cartilage degeneration [3]. On the opposite, physical activity (PA) practiced by young adults is positively associated with cartilage volume, and this seems protective against the development of osteoarthritis (OA) [4]. Fortunately, cartilage deforms during physiological activities and usually recovers after loading [5], making physical exercise ideal to increase blood flow to the connective tissues of the joints and prevent cartilage deterioration.

Even if the literature suggests positive outcomes of exercise in young adults [4], the effects of PA in adults and older adults are contradictory. Some studies suggest that high PA levels do not damage the cartilage of the knee [6] also if practiced for years [7], and it is suggested that it could reduce cartilage loss [8], slowing the progression of cartilage degeneration [3]. Differently, other studies suggest that PA has a deleterious effect on cartilage [9] causing degeneration [10] or worsening the composition [11], and accelerating the disruption of joint stability [3]. Higher PA levels may be at greater risk for cartilage, meniscus and ligament abnormalities [12].

These contradictory ideas on the effects of PA on cartilage should be due to the different dosages of the interventions, indeed, an underload or an overload are associated with cartilage damage [13]. Consequently, it is important the development of a PA intervention that includes aerobic and muscle resistance exercises to protect joints, promote health, and reduce disability in older age [14]. The importance of understanding the effects of a structured PA program increases in people with mild to moderate OA; indeed, the intervention should have to reduce pain and disability levels [15] not presenting deleterious effects.

The so-called Standard Operating Procedures are being widely adopted in other fields to proceed correctly and limit possible errors [16]. Their use could help standardize the interventions concerning PA in OA individuals and foster the clarification of the PA preventive and protective role towards cartilage health. The objective of the present review is to understand how different PA typologies interventions affect cartilage; and then propose a Standard Operating Procedure for an exercise program to prevent joint cartilage degeneration and maintain knee joint health, especially during aging.

## 2. Materials and Methods

This systematic review and meta-analysis follows the principles outlined by the PRISMA guidelines [17], and the checklist can be found in the Supplementary Materials. The protocol is not registered in a specific database, but it was written before the systematic review performance.

### 2.1. Eligibility Criteria

Population, Intervention, Comparison, Outcomes, and Study (PICO-S) criteria are followed. The population of interest is composed of people aged 18 years and older, both males and females, and no restrictions were adopted on body composition. Participants were excluded if they were professional or elite athletes; or if they presented physical (i.e., muscular dystrophy, hypotonia, injury to tendons or ligaments) or movement disorders (i.e., akinesia, bradykinesia, or dystonia). For the intervention, manuscripts are included if PA training is proposed without any other complementary intervention, to limit the confounding factors. Consequently, studies were excluded if presented with other complementary factors that could influence cartilage. The comparators considered are the cartilage evaluation pre- and post-intervention. Outcomes considered are related to magnetic resonance imaging (MRI) acquisition and blood and urinary samples. Only English written, original, peer-reviewed articles that adopted cross-sectional, correlational, randomized, and nonrandomized controlled, as well as quasi-randomized studies are included (study design). Other studies’ design typologies were excluded.

### 2.2. Data Collection

The systematic search includes studies published until the 20 January 2022 (the day of the search) performed through the electronic databases PubMed, Web of Science, and Scopus. The following keywords were adopted:

Keywords 1: articular cartilage; fibrocartilage; gristle.

Keywords 2: exercise; physical activity; fitness; movement; sport.

The keywords were matched adopting the Boolean operators OR/AND, and the following string has been created:

(“Articular Cartilage” OR fibrocartilage OR gristle) AND (exercise OR “physical activity” OR fitness OR movement OR sport).

### 2.3. Study Record

EndNote software (EndNote version X8; Thompson Reuters, New York, NY, USA) was adopted to identify duplicates. The selection process was performed by two independent investigators, who screened the titles first, then the abstracts, and the full-length articles. In case of disagreement in categorizing a manuscript, the principal investigator considered the studies independently and provided the tie-breaking decision. Investigators were not blinded to the study title, authors, or associated institutions during the selection process.

### 2.4. Data Extraction and Analysis

A Microsoft Excel^®^ (Microsoft Corp., Redmond, WA, USA) spreadsheet was compiled with the following information: first author; year of publication; type of study; sample size; participants’ age (range or mean ± SD); gender; intervention characteristics; and outcomes. The main outcomes are discussed narratively.

### 2.5. Quality and Risk of Bias Assessment

Selection bias was analyzed by dividing the participants of the studies in groups based on age and health status. The studies were divided based on their design, and the randomized controlled trials were evaluated by two investigators through the PEDro scale, a third investigator was involved only in case of disagreement. This scale is composed of 11 items [18], and a score between 0 to 10 (high quality: excellent (9–10 points), good (6–8 points), fair (4–5 points), and poor (less than 4 points) quality. This scale presents good reliability [18] and validity [19].

## 3. Results

**Figure 1:**
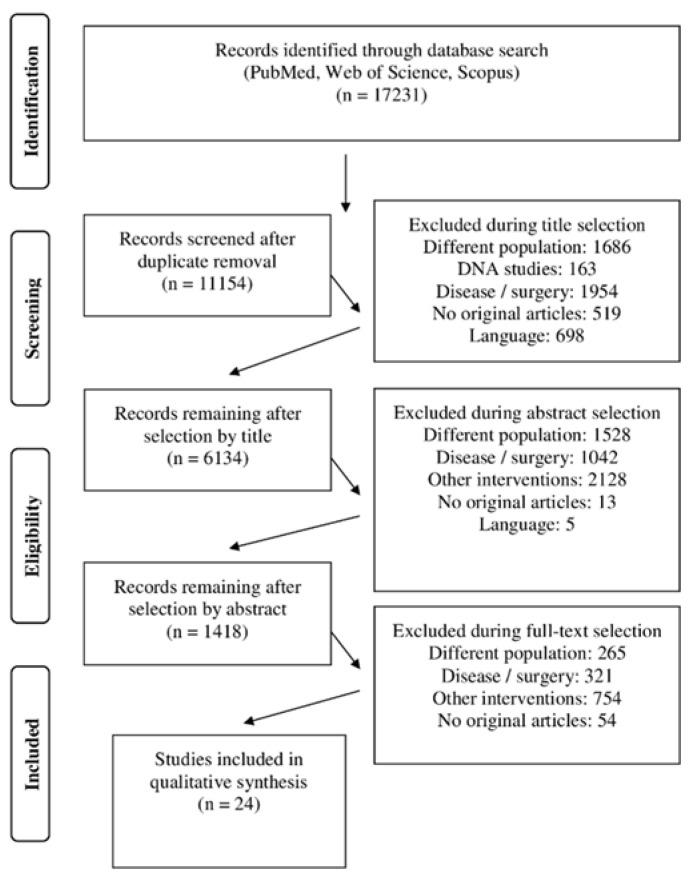
Flow chart of the study collection process.

From a total number of 17,231 collected after the electronic search, 11,154 is the number of studies left after the duplicate’s removal. After the screening process, the final number of included studies is 24 studies. The flow chart is presented in Figure 1.

A total of 1222 (age range 20–70) participants, 748 of them with OA, have been included. The mean age was 47 (18), 542 were females, 380 were males; for 300, the gender was not specified. The main study characteristics are summarized in Table 1.

### 3.1. Characteristics of the Included Studies

The intervention ranges from 7 days to 120 weeks, with four studies that propose 12 weeks. The frequency ranges from one to five times per week (the most adopted is three times a week). The session duration ranges from 30 to 90 min. Eight interventions study the acute effects of PA on cartilage. Eight studies propose a supervised intervention, at least in the first period during which participants learned the exercise routine. Six studies move the intervention to participants’ homes making the intervention possible outside the gym. Some of the studies evaluate the effects of amatorial sports practice such as swimming (*n* = 1), running (*n* = 7) and cycling (*n* = 3).

Related to the association between exercise training, pain, and functional outcomes, only 10 studies provided data pre- and post-intervention. Azukizawa et al. [20], Centeno et al. [24], Mikesky et al. [36], and Multanen et al. [37] generally detected no significant changing after physical exercise related to pain and functional outcomes. Dinçer et al. [25], Ikuta et al. [31], Kangeswari et al. [32], Knoop et al. [35], and Vassao et al. [41] detected better results after a physical exercise treatment both for the pain scale and functional outcomes.

### 3.2. Example of Interventions

Well-rounded exercise programs include stretching, balance exercises, aerobic activity, functional strengthening, and resistance exercises [20,24]. The body alignment of the core, pelvis and low extremities, and a balance/neuromuscular training could help the interventions and enhance the results [24].

Specific muscle strengthening interventions [25,28,31,35,36,41] and isometric exercises are proposed [32]. One of the intervention proposals is individually progressed and consisted of 5 min warm-up and hopping (3–5 sets, 15 s of rest between sets) exercises of approximately 3–4 min [28]. Resistance training interesting leg press, leg curl, seated chest press, and seated back row, upper body exercises [36], or bodyweight squat exercises [25] are also proposed. Similarly, an intervention proposes muscle strengthening exercises for the gluteal area, hamstrings, quadriceps, and triceps surae muscles [31]. Another intervention consists of 5 min on treadmill, six strength exercises (3 sets; 8 repetitions; 60% of 1-RM; rest interval of 2–3 min: hip abductors and adductors chair, seated leg raise, glute bridge, knee flexors and extensors chair), and stretching of major muscle groups [41]. The interventions could be supported also by knee joint stabilization exercises [35].

Related to cardiovascular interventions, aerobic exercise includes high-impact loading (jumping exercises) and rapid change of direction with music [37,38]. Other aerobic activities consisted of amateur swimming, running, and cycling (60–70% of their heart rate) [23]. More information related to the intervention’s proposal are provided in Table 2.

### 3.3. Quality and Risk of Bias Assessments

The PEDro scale for the risk of bias and quality of the studies assessment for the randomized controlled trails of the included studies presents a good quality with a mean range score of 6.8. It ranged from 7 to 10. Results are summarized in Table 3.

## 4. Discussion

Generally, PA has positive outcomes on cartilage structure regardless of the duration, frequency, intensity, and type of the intervention, suggesting positive effects due to an active lifestyle also during elderly. In this review, we highlighted the different effects of PA on the health of cartilage; however, attention in the field is required because all the considered interventions adopted different and not structured protocols. A guideline to structure the intervention is required; therefore, we propose the Standard Operating Procedure to correctly address the intervention for cartilage health. It is important to consider our standard operating procedure proposal is always based on the cartilage health status of the people trained.

The Standard Operating Procedure includes aerobic, resistance and stretching exercises. Aerobic capacity and resistance training should be proposed on different days [43]. Flexibility is part of the intervention, postural balance and coordination should be added to obtain the best results [20], also in people with severe OA [44]. If the individual is overweight, it is important to include weight loss in the intervention [45]. Furthermore, if it is present knee malalignment, the intervention should include also exercises correcting the angle of the knee during gait to reduce the degeneration, especially of the posteromedial knee [31]. Ideally, the minimum length of the intervention should be of 12 weeks with a frequency of 3 times a week for 60 min each session. In the first week of the intervention, people should be adequately prepared by trainers to correctly perform the exercise routine without supervision. After this learning period, people should perform the exercises independently at home and throughout their life. The Standard Operating Procedure is presented in Table 4.

Walking is the simplest aerobic activity without adverse effects on articular cartilage metabolism [21]; indeed, there is no association between daily walking and structural changes over two years in people at risk of or with mild knee OA [46]. If walking is not sufficient, also running is a suitable intervention, the only precaution is that people with OA require longer recovery times [26]. Another precaution to consider when running is to increase the step rate frequency that decreases cartilage contact area, impact peak and cartilage contact pressure [47]. Running produces better results than swimming or cycling by inducing an impact on the cartilage that consolidates articular-cartilage tissue [23] making it ideal as an aerobic activity. Exercise in water presents good short-term effects but it appears deleterious in the long term [45,48]. Literature suggests that high-impact exercises do not negatively affect knee cartilage biochemical composition or knee pain [37]; so it is important to include this stimulus in the PA intervention.

Similar to aerobic intervention, resistance exercises have to be included in the ideal Standard Operating Procedure [46] also in people with advanced OA [35]. Isometric exercise reduces pain, stiffness, and physical dysfunction [32] with a beneficial effect on the cartilage [20]. Exercises to strengthen the quadriceps and hamstring muscles have no adverse effect on knee cartilage volume and thickness, reduce the pain and increase functionality [25]. Clinicians have to be aware that when alterations in the normal knee physiology are present, aerobic activities should be limited (no more than 10,000 steps/day), increasing weight-bearing activities to maintain PA level [49]. In order to measure the daily activities, individuals with OA could use smartwatches or fitness trackers to collect the PA during the day including both walking and exercising [50].

All grades of OA severity can benefit from PA, although effects might be reduced in patients with advanced OA [35]. It is important to propose a structured intervention especially for those people with OA because exercise reduces pain and disability [24,51]. On the opposite, sports prone to joint injury or vigorous high-impact should be avoided because they present an increased risk for OA [52]. Moderate to strenuous exercise and frequent knee-bending activities may accelerate cartilage degeneration and abnormalities [53].

Data on the mouse model suggest that moderate PA improves tribology and lubricative properties of articular cartilage, promoting lubricin synthesis and its elevation in synovial fluid, thus preventing cartilage degradation [54,55,56,57]. Consequently, PA, whatever the training, has benefits and can be considered a complementary and optional primary treatment for OA [58]. Regular moderate impact exercise does not increase the risk of OA, and there is some evidence that it does not increase symptoms in patients with mild OA [59]. Physical activity may not have a detrimental effect on the knee joint and thus be beneficial to joint health [60], suggesting this intervention as a preventive approach for cartilage degeneration.

The first limitation of the present study was related to the heterogeneity in the information provided about the health of the cartilage of the participants included. Furthermore, studies that included diet or other interventions proposed in a complementary way were not considered probably excluding some studies with interesting PA interventions. This decision was taken to limit the confounding factors. Another important limitation of the study it has been the impossibility to perform a meta-analysis due to the differences in the population (healthy people vs. people vs. OA with different gender and age), intervention (acute vs. long term effects), comparison (blood samples, MRI, other variables), and outcomes (knee or hip articulation, vertebrae). Future studies should investigate the effects of PA on people with different gravity in cartilage damage and determine the proper cut-off for the practice of the most appropriate PA typology. Second, studies should have to investigate accurately the interaction of different sports and their effects in the presence of OA. For instance, sports such as enduro motorcycling, where the individual is exposed to prolonged vibrations, may predispose the individual to early OA degeneration [61].

## 5. Conclusions

Structured PA interventions have mainly positive outcomes on cartilage structure. Because the interventions were different and various a Standard Operating Procedure has been proposed for a physical intervention focalized on cartilage wellness. Clinicians and researchers have to be aware when prescribing PA programs to select the most appropriate intervention based on the patient’s needs.

This study wants to provide the community with guidelines to prevent or limit cartilage degeneration and guarantee a healthy and active older age. Furthermore, a proper and accurate method to monitor the interventions is required to supervise the PA intervention and its outcomes. Finally, non-invasive techniques could help the researcher to detect early cartilage degenerations and plan structured PA programs personalized to the patients’ needs.

## Figures and Tables

**Table 1:** Overview of the studies characteristics.

1st Author	Study Design	Pop	Number (m)	Age (sd)	Method	Biomarkers	Conclusions
Azukizawa 2019 [20]	IS	OA	42 (0)	59 (6)	blood and urine sample ELISA	sPiiCP; uCtX-ii; uC2C; sCOMP	Well-rounded exercise improves PA and has beneficial effects on type 2 collagen metabolism
Bautch 2000 [21]	RCT	OA	21 (7)	70 (2)	synovial fluid (knee joints) ELISA	chondroitin sulphate epitopes 3B3, 7D4, GAG	No deleterious effects on osteoarthritic joints and ameliorated joint pain
Boocock 2009 [22]	IS	H	20 (10)	33 (9)	MRI	Cartilage volume	Running resulted in deformation of femoral, medial, and lateral tibial articular cartilage volume.
Celik 2013 [23]	RCT	H	44 (44)	22 (2)	blood samples ELISA	sCOMP	Regular, weight-bearing, high-impact physical exercise consolidates cartilage tissue
Centeno 2018 [24]	RCT	OA	48	55 (9)	none	none	PA is an effective alternative therapy for KOA
Dinçer 2016 [25]	RCT	OA	30 (6)	51 (5)	MRI	Cartilage volume	No significant effect of closed kinetic chain exercise on the cartilage volume or morphology.
Esculier 2019 [26]	Pilot study	OA	20 (0)	52 (8)	MRI	T2_s	No changes after 30 min of running. People with KOA need more time to recover
Gatti, 2017 [27]	IS	H	15 (15)	26 (4)	MRI	T2_s, cartilage volume	Run shortened tibiofemoral cartilage T2, not bike
Hartley 2019 [28]	RCT	H	42 (0)	55–70	MRI	T2_s	A high-impact exercise intervention has no negative effects on KOA.
Helmark 2010 [29]	RCT	OA	29 (19)	66 (6)	blood and urine samples ELISA	COMP, Aggrecan, CTX-II, IL6, IL8, IL10; TNF-α.	Positive effect of PA on a chondroprotective anti-inflammatory cytokine response in KOA
Horga 2020 [30]	PS	H	44 (17)	45	MRI	Lesions	The knees achieved sustained improvement, for at least 6 months post-marathon
Ikuta 2020 [31]	RCT	OA	26 (3)	68 (9)	MRI	T2_m	PA could be a treatment to improve the course of KOA
Kangeswari 2021 [32]	RCT	OA	200	45–65	none	none	Isometric exercise program reduce pain, stiffness and improve physical function in KOA
Kessler 2020 [33]	PS	H	19 (10)	30 (6)	MRI	T2_s	Joint-loading with a stepping activity resulted in T1ρ and T2 changes above background measurement error
Kingsley 2012 [34]	IS	H	8 (8)	21 (1)	MRI	T2_s, Cartilage volume	Changes were observed throughout the thoracic and lumbar vertebral regions
Knoop, 2014 [35]	RCT	OA	95 (31)	61 (7)	MRI	Cartilage lesions	Effectiveness of PA is independent of OA severity
Liangyu 2014	RCT	H	120	42	MRI	Cartilage volume	Decrease the total knee cartilage volume
Mikesky 2006 [36]	RCT	H	221 (93)	69	radiographic evaluation	OA severity	Strength training retained more strength and exhibited less frequent progressive joint space narrowing
Multanen 2014 [37]	RCT	OA	80 (80)		MRI	T2_s	PA improve balance, force, and endurance. No effect on cartilage
Multanen 2017 [38]	RCT	OA	78 (0)	58 (4)	MRI	T2_s	High-impact training increase femoral neck strength not affecting knee cartilage on KOA
Pruksakorn 2013 [39]	IS	H	82 (32)	20	blood sample; ELISA	COMP, WF6, HA	Articular cartilage is susceptible to the increasing load
Subburaj 2012 [40]	IS	H	20 (10)	29	MRI	T2_s, Cartilage volume	Acute effect of run on knee cartilage and meniscus composition
Vassao 2021 [41]	RCT	OA	23	64 (4)	Blood sample ELISA	IL6, IL8, IL10, IL1β, TNF-α	Physical exercise increases the functional capacity
Yanagisawa 2021 [42]	IS	H	15 (11)	23 (3)	MRI	Apparent Diffusion Coefficient	High-load deadlift exercise stress the lumbar intervertebral discs

Note: chondroitin sulfate-WF6: WF6; Enzyme-linked immunosorbent assays: ELISA; healthy: H; hyaluronic acid: HA; knee osteoarthritis: KOA; Interleukin: IL; intervention study: IS; magnetic resonance imaging: MRI; osteoarthritis: OA; physical activity: PA; prospective study: PS; population: Pop; randomized controlled trial: RCT; serum cartilage oligomeric matrix protein: sCOMP; serum cartilage type ii procollagen carboxy propeptide: sPiiCP; sulphated glycosaminoglycan: GAG; Transverse relaxation time: T2; T2 maps (non-contrast compositional MRI): T2_m; T2 MRI sequence; T2_s; Tumor Necrosis Factor: TNF-α; urine C-terminal telopeptide of collagen type ii: uCtX-ii; urine cleavage of type ii collagen by collagenases: uC.

**Table 2:** Characteristics of the interventions adopted in the included studies.

1st Author, Year	Length (Weeks)	Frequency (Days a Week)	Duration (Minutes)	Tutoring	Intervention
Azukizawa 2019 [20]	12	1	90	supervised them HB	stretching, balance, walk, and isometric exercises
Bautch 2000 [21]	1	3	60	NI	strengthening; low-intensity walking
Boocock 2009 [22]	AE	NI	NI	NI	run
Celik 2013 [23]	12	3	40	NI	swimming, running, cycling. 60–70% of heart rate
Centeno 2018 [24]	NI	NI	NI	HB	strengthening, resistance, functional, balance/neuro-muscular, aerobic, ROM
Dinçer 2016 [25]	2	5	30	NI	strengthening
Esculier 2019 [26]	AE	NI	NI	NI	run
Gatti, 2017 [27]	AE	NI	NI	NI	bike and run
Hartley 2020 [28]	24	NI	50 hops	NI	high impact exercise progressing
Helmark 2010 [29]	AE	NI	NI	NI	resistance training
Horga 2020 [30]	28	NI	NI	NI	run
Ikuta, 2020 [31]	NI	NI	NI	supervised them HB	ROM and muscle strengthening; leg stretching
Kangeswari 2021 [32]	12	3	40	supervised then HB	isometric exercises
Kessler 2020 [33]	AE	NI	NI	NI	step
Kingsley, 2012 [34]	AE	NI	NI	NI	walk
Knoop, 2014 [35]	12	2	60	supervised and HB	knee joint stabilization muscle strengthening
Mikesky, 2006 [36]	120	3	NI	supervised then HB	resistance training
Multanen 2014 [37]	48	3	55	supervised	aerobic and step-aerobic jumping exercise
Multanen 2017 [38]	48	3	55	supervised	high-impact aerobic and step aerobic
Pruksakorn 2013 [39]	AE	NI	NI	NI	walk
Subburaj 2012 [40]	AE	NI	NI	NI	run
Vassao, 2021 [41]	8	2	NI	supervised	strength exercises and stretching
Yanagisawa 2021 [42]	AE	NI	NI	NI	stretching and submaximal deadlift repetitions

Acute effect: AE; Home base: HB; no info: NI; repetition maximum: RM.

**Table 3:** PEDro scale for the risk of bias and quality of the studies assessment for the randomized controlled trails manuscript included.

Author	1	2	3	4	5	6	7	8	9	10	11	10/10
Bautch 2000 [21]	1	1	0	1	0	0	0	1	0	1	1	5/10
Celik 2013 [23]	1	1	1	1	1	1	1	1	0	1	1	9/10
Centeno 2018 [24]	1	1	1	1	0	0	0	0	0	1	1	5/10
Dinçer 2016 [25]	1	1	0	1	0	1	1	1	0	1	1	7/10
Hartley 2019 [28]	1	1	0	1	0	0	1	0	0	1	1	5/10
Helmark 2010 [29]	1	1	0	1	0	0	0	1	0	1	1	5/10
Ikuta 2020 [31]	1	1	0	1	0	0	0	1	0	1	1	5/10
Kangeswari 2021 [32]	1	1	0	1	1	1	1	1	0	1	1	8/10
Knoop 2014 [35]	1	1	1	1	0	0	1	1	0	1	1	7/10
Mikesky 2006 [36]	1	1	1	1	1	0	1	1	0	1	1	8/10
Multanen 2014 [37]	1	1	1	1	0	1	1	1	0	1	1	8/10
Multanen 2017 [38]	1	1	0	1	0	0	1	1	0	1	1	6/10
Vassao 2021 [41]	1	1	0	0	1	1	0	1	0	1	1	6/10
Total												6.8/10

Note: 1. Eligibility criteria (unscored); 2. Subject random allocation; 3. Allocation concealed; 4. The groups were similar at baseline; 5. Subject blinded; 6. Therapists blinded; 7. Assessors blinded; 8. more than 85% retention rate; 9. Intention to treat; 10. Between-group analysis; 11. Point measures and measures of variability of at least one key outcome.

**Table 4:** Standard Operating Procedure proposal for an intervention for the cartilage wellness.

Training/Intervention	Aerobic intervention/walking-running
	Strength/isometric exercise
	Flexibility/all major muscles
	Person-specific necessity: postural balance; knee alignment; loss of weight
Frequency	3 times a week
Duration	60 min
Intensity	Gradually increased
Supervision	Only in the first period. It is suggested that the intervention become a home-based exercise

## Data Availability

Not applicable.

## Supplementary Materials

The following supporting information can be downloaded at: https://www.mdpi.com/article/10.3390/healthcare10101821/s1. PRISMA checklist.
